# Evaluation and Determination of Quantitative Hepatitis B Surface Antigen Diagnostic Performance in Chronic Hepatitis B Virus-Infected Patients

**DOI:** 10.7759/cureus.41202

**Published:** 2023-06-30

**Authors:** S M Rashed Ul Islam, Umme Shahera, Munira Jahan, Shahina Tabassum

**Affiliations:** 1 Virology, Bangabandhu Sheikh Mujib Medical University, Dhaka, BGD

**Keywords:** diagnostic performance, receiver operating characteristics (roc), hbv-dna, qhbsag, chb patients

## Abstract

Background

Hepatitis B virus DNA (HBV-DNA) assessment is recommended for diagnosing and monitoring chronic hepatitis B (CHB) patients. Quantitative hepatitis B surface antigen (qHBsAg) estimation adjunct to HBV-DNA is vital for assessing HBV chronicity and therapeutic prognosis. This study aimed to estimate the qHBsAg and compare its diagnostic performance with that of the HBV-DNA levels in CHB patients from Bangladesh.

Methodology

A total of 148 CHB patients were enrolled in this study. qHBsAg and hepatitis B e-antigen (HBeAg) were estimated using chemiluminescent and enzyme immunoassays, respectively, and HBV-DNA was quantified using real-time polymerase chain reaction. The parameters and diagnostic performances were analyzed by receiver operating characteristic (ROC) curve analysis.

Results

The overall levels (mean ± SD) of qHBsAg, HBV-DNA, and alanine aminotransferase (ALT) among the total population (n = 148) were 3.45 ± 0.80 log_10_ IU/mL, 4.40 ± 2.44 log_10_ IU/mL, and 86.17 ± 39.06 IU/L, respectively. Significant differences were observed in the levels of both qHBsAg (p = 0.004) and HBV-DNA (p < 0.0001) in cases with HBeAg positivity. qHBsAg levels showed a weak positive correlation with the levels of HBV-DNA and ALT in HBeAg-positive CHB patients, but no such relationship was observed in HBeAg-negative CHB patients. ROC curve analysis showed that the area under the curve for the qHBsAg level to distinguish high HBV-DNA levels (>5 log_10_ IU/mL) was 0.653 (p = 0.002), which indicated an acceptable diagnostic performance. The best cut-off of qHBsAg for predicting high HBV-DNA levels was 3.469 log_10 _IU/mL.

Conclusions

Our results indicated that qHBsAg might be a useful marker for monitoring HBV-DNA in CHB patients throughout treatment and follow-up.

## Introduction

Infection with the hepatitis B virus (HBV) leads to a wide spectrum of liver illnesses ranging from acute to chronic hepatitis, cirrhosis, and hepatocellular carcinoma (HCC) [1]. Serological marker detection is the backbone for diagnosing and confirming HBV infection, and hepatitis B surface antigen (HBsAg) in serum has been considered the most reliable marker of HBV infection [2]. The hallmark of chronic hepatitis B (CHB) infection is the presence of HBsAg seropositivity for at least six months [3]. In addition to HBsAg, the measurement of HBV-DNA is another useful marker to evaluate CHB infection [4]. HBV-DNA measurement at three- to six-month intervals is crucial for detecting viral replication activity [5] and for monitoring the virus’ suppression in patients receiving treatment [6]. Both qHBsAg and HBV-DNA provide different but similar information that might help clinicians understand the CHB virus infection status [7]. HBV-DNA is synthesized via the reverse transcription of pre-genomic RNA derived from covalently closed circular DNA (cccDNA). HBsAg is also one of the byproducts of cccDNA transcription in hepatocytes. Therefore, cccDNA is the common template for both HBsAg and HBV-DNA synthesis [8]. cccDNA measurement in liver tissue indicates the actual infection severity of hepatocytes, but routine use of the test is limited by its complexity [9]. Furthermore, it is challenging to measure cccDNA because only liver biopsy allows for cccDNA detection. On the other hand, qHBsAg acts as the most approachable marker that can reflect the level of transcriptional activity of cccDNA [10]. Thus, the focus has been transformed toward the qHBsAg due to its association with the level of cccDNA, which reflects the viral replication activity inside the nuclei of hepatocytes [11].

Over recent years, qHBsAg has been reported to show a good correlation with transcriptionally active cccDNA. It has also attracted much attention due to its ability to stratify the risk of disease progression [7]. Automated quantification of qHBsAg has been developed globally at low cost and duly standardized with the universal unit of IU/mL [12]. Various studies have demonstrated the clinical utility of qHBsAg and HBV-DNA quantification; however, there has been no study from Bangladesh in which these two markers have been evaluated and compared. Therefore, the present study compared qHBsAg and HBV-DNA and evaluated the diagnostic efficacy of qHBsAg levels in the CHB virus-infected Bangladeshi population.

This article was previously presented as a meeting abstract at the VIROCON 2018, the 27th International Conference on “Global Viral Epidemics: A Challenging Threat” from November 12th to 14th, 2018.

## Materials and methods

Ethical considerations

Before starting research activities, this project received Institutional Review Board (IRB) approval from Bangabandhu Sheikh Mujib Medical University (BSMMU/2017/12723 on December 20, 2017). The study adhered to the ethical principles established at the 64th World Medical Association General Assembly held in Fortaleza, Brazil, in October 2013.

Patients and study design

The presented cross-sectional study included a total of 148 CHB virus-infected patients who attended the Department of Virology, BSMMU for their HBV-related virological investigation from January 2018 to December 2018. The patients’ demographic characteristics were reviewed using the clinical data repository of the departmental information system. Informed consent was obtained from all participants. About 10 mL of whole blood samples were collected and processed for further laboratory procedures while maintaining universal biosafety precautions.

Laboratory analysis

Serological detection of qHBsAg and hepatitis B e-antigen (HBeAg) was done by a chemiluminescent immune assay and automated enzyme-linked immunosorbent assay technique, respectively. HBV-DNA load was measured by a real-time polymerase chain reaction (PCR) system, and alanine aminotransferase (ALT) was quantified using the International Federation of Clinical Chemistry method. qHBsAg levels were measured by the LIAISON XL murex HBsAg Quant (DiaSorin) assay per the manufacturer’s protocol and the results were expressed as IU/mL. A patient with HBsAg >0.050 IU/mL was considered HBsAg-positive. This assay was calibrated against the Second International Standard for HBsAg, subtype adw2, genotype A, NIBSC code 00/588, which allowed the quantitation of HBsAg within the range of 0.030 to 150 IU/mL. The exact concentration of qHBsAg was assessed after samples with qHBsAg >150 IU/mL were diluted at a 1:500 ratio with the recommended diluent provided by the manufacturer. HBV-DNA quantification was done using the Applied Biosystems 7500 real-time PCR system per the manufacturer’s protocol described elsewhere [13]. HBV-DNA load was expressed as IU/mL. ALT level (IU/L) was measured using the semi-automated bioanalyzer using Meriline-SGPT (Meril Diagnostics, India).

Statistical analysis

The HBeAg serological status was expressed as frequency (n) and percentage (%), and the levels of qHBsAg, HBV-DNA, and ALT were expressed mean ± standard deviation (mean ± SD). Both qHBsAg and HBV-DNA levels were transformed to log_10_ for a presentation where necessary. An independent t-test was done to compare continuous variables, and Pearson’s correlation (r) coefficient was used to determine the correlation of qHBsAg with both HBV-DNA and ALT levels. The determination of the best cut-off point for high HBV-DNA load (>5 log_10_ IU/mL) was done using receiver operating characteristic (ROC) >5 log10 IU/mL analysis. Data analysis was done using SPSS software for Windows version 19 (IBM Corp., Armonk, NY, USA). P value ≤0.05 was considered statistically significant.

## Results

Out of 148 enrolled participants, 109 (73.6%) were male, while 39 (26.4%) were female. The participants’ average (mean ± SD) age was 31.50 ± 12.43 years (range = 8-76 years). Among the study population, 68 (45.9%) were HBeAg-positive, while 80 (54.1%) were HBeAg-negative. The overall mean levels of qHBsAg, HBV-DNA, and ALT were 3.45 ± 0.80 log_10_ IU/mL, 4.40 ± 2.44 log_10_ IU/mL, and 86.17 ± 39.06 IU/L, respectively. The detailed characteristics of the study population are described in Table 1. Independent t-test revealed that both qHBsAg (p = 0.004) and HBV-DNA (p < 0.0001) values differed significantly between HBeAg-positive and HBeAg-negative CHB patients; however, ALT values between the groups were not significantly different (p > 0.05).

**Table 1:** Characteristics of the study population. Data are presented as mean ± SD or n (%). qHBsAg = quantitative hepatitis B surface antigen; HBV-DNA = hepatitis B virus DNA; ALT = alanine aminotransferase; HBeAg = hepatitis B e-antigen

Variables		Overall	HBeAg-positive	HBeAg-negative	P-value
Gender	Male	109 (73.6%)	50 (73.5%)	59 (73.8%)	>0.05
	Female	39 (26.4%)	18 (26.5%)	21 (26.3%)	>0.05
Age (in years)		31.50 ± 12.44	29.69 ± 12.95	33.04 ± 11.84	>0.05
qHBsAg (log_10_ IU/mL)		3.45 ± 0.80	3.66 ± 0.77	3.28 ± 0.79	0.004
HBV-DNA (log_10_ IU/mL)		4.40 ± 2.44	6.34 ± 1.73	2.77 ± 1.60	<0.0001
ALT (IU/L)		86.17 ± 39.06	82.81 ± 31.76	89.03 ± 44.34	>0.05

Overall, the study population exhibited a weak but significant connection between qHBsAg and HBV-DNA (r = 0.29; p < 0.0001). Although there was no significant association between qHBsAg and HBV-DNA in the HBeAg-negative patients, there was a significant positive correlation in the HBeAg-positive CHB patients (r = 0.394; p = 0.001). Similarly, among all CHB patients, a weak but significant correlation was found between qHBsAg and ALT (r = 0.171, p = 0.038). A significant positive correlation was observed between qHBsAg and ALT in HBeAg-positive patients (r = 0.247, p = 0.042); however, these factors did not show any significant correlation in the case of HBeAg-negative CHB cases (r = 0.168, p = 0.135). The correlation analysis is presented in Table 2.

**Table 2:** Correlation of qHBsAg with HBV-DNA and ALT based on the HBeAg seropositivity. qHBsAg = quantitative hepatitis B surface antigen; HBV-DNA = hepatitis B virus DNA; ALT = alanine aminotransferase; HBeAg = hepatitis B e-antigen

Variables		n	r	P-value
HBV-DNA	Overall	148	0.290	<0.0001
	HBeAg-positive	68 (45.9%)	0.394	0.001
	HBeAg-negative	80 (54.1%)	-0.51	>0.05
ALT	Overall	148	0.171	0.038
	HBeAg-positive	68 (45.9%)	0.247	0.042
	HBeAg-negative	80 (54.1%)	0.168	>0.05

**Figure 1:**
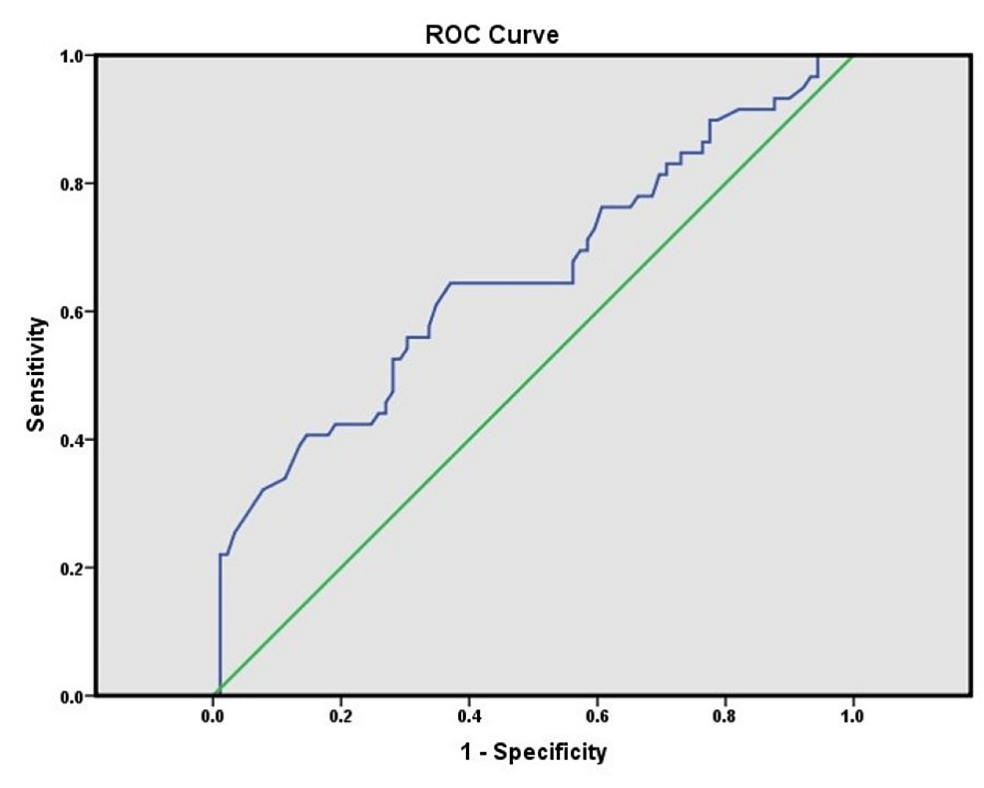
ROC curve analysis of qHBsAg for the detection of high HBV-DNA load in CHB-infected patients. qHBsAg = quantitative hepatitis B surface antigen; HBV-DNA = hepatitis B virus DNA; ROC = receiver operating characteristic; CHB = chronic hepatitis B

A ROC curve analysis was performed to identify the best qHBsAg cut-off point to characterize a high HBV-DNA load (>5 log_10_ IU/mL). According to the ROC curve, the area under the curve (AUC) for qHBsAg level was 0.653 (95% CI = 0.560-0.746; p = 0.002) when determining high HBV-DNA level. The best cut-off qHBsAg level for predicting high DNA level was found to be 2.95 × 10^3^ IU/mL (3.469 log_10_ IU/mL). However, we did not obtain any significant AUC value of qHBsAg that reflected a cut-off for predicting lower HBV-DNA levels (Figure 1).

## Discussion

Estimating qHBsAg and HBV-DNA can provide insights into the evaluation of HBsAg seroclearance. qHBsAg level varies depending on the balance between viral replication and host immunity [14]. qHBsAg values indirectly reflect the number of infected hepatocytes and their fluctuations during the natural progression of CHB infection [15]. Different studies have reported that HBeAg-positive CHB patients exhibit higher qHBsAg and HBV-DNA load than HBeAg-negative CHB cases [16]. Similar patterns of alteration in both qHBsAg and HBV-DNA levels were also observed in this study.

In HBeAg-negative patients, patients with HBV-DNA >2,000 IU/mL and patients with advanced liver fibrosis have a high risk of disease progression and HCC. It has been observed that, in patients with HBV-DNA <2,000 IU/mL and without liver fibrosis, qHBsAg <1,000 IU/mL signifies an inactive carrier state with low HCC risk, and HBsAg <100 IU/mL is linked to a significant rate of spontaneous HBsAg seroclearance [17]. Thus, qHBsAg and HBV-DNA are vital for CHB patient monitoring and early prognosis determination. This study observed a significant correlation between qHBsAg and HBV-DNA levels. Our results also showed that there was a stronger correlation between qHBsAg and HBV-DNA levels in HBeAg-positive CHB patients, but no correlation was observed in HBeAg-negative CHB patients. An earlier study [11,18] supports this observation. In another study, no significant correlation was observed between qHBsAg and HBV-DNA in both HBeAg-positive and negative patients (r = 0.309, p = 0.093; r = 0.065, p = 0.443; respectively) [19]. On the contrary, an earlier Korean study revealed a weak but significant positive correlation between qHBsAg and HBV-DNA; however, this relationship was not observed during the late replicative phases of CHB infection [20].

High levels of qHBsAg (>5 log_10_ IU/mL) and HBV-DNA (>8 log_10_ IU/mL) were shown as a hallmark of the immune-tolerant phase in HBeAg-positive Asian carriers [21]. Accordingly, qHBsAg levels of 4.397 log_10_ IU/mL and normal ALT levels in CHB patients were associated with low fibrosis in 140 Chinese HBeAg-positive carriers [22]. Hence, HBV-DNA, ALT, and HBeAg levels are the most important risk assessment markers of progression to cirrhosis that lead to HCC development [4,23].

A previous study reported higher ALT, aspartate transaminase, and bilirubin levels in HBeAg-negative CHB patients compared to HBeAg-positive CHB patients [24]. On the contrary, a Chinese study showed higher levels of ALT in HBeAg-positive CHB patients than in HBeAg-negative CHB patients [25]. Here, we observed a weak positive correlation of qHBsAg with HBV-DNA and ALT in the study sample; however, the results were inconclusive. Thus, this study has highlighted that qHBsAg is not a viable substitute for HBV-DNA and ALT. Instead, it gives complementary information and must be interpreted along with other viral parameters in different clinical settings [17].

The European Association for the Study of the Liver, 2017 (EASL, 2017) guidelines mention that the determination of HBsAg levels can help in determining the frequency of follow-up in such patients [26]. Patients with HBsAg levels <1,000 IU/mL can be followed for ALT levels every 12 months and HBV-DNA and liver fibrosis assessments every three years, while patients with HBsAg levels ≥1,000 IU/mL should be followed for ALT levels every six months and HBV-DNA and liver fibrosis assessments at least every two years [27]. The ROC curve was plotted to determine whether the qHBsAg level could significantly differentiate the HBV-DNA levels in the CHB patients.

We attempted to suggest a cut-off value of qHBsAg titer that will best match high HBV-DNA values to predict the high replicative status of CHB patients. The qHBsAg cut-off value found in this study was nearly similar to that shown in an earlier Saudi Arabian study [28]. Another study from India showed that the cut-off value of qHBsAg was 3.526 log_10_ IU/mL to detect HBV-DNA >2,000 IU/mL [29]. The qHBsAg values in all the above-mentioned studies were found to be fairly similar to those in the present study.

Study limitations

This study has attempted to determine the relationships between qHBsAg and other viral markers of CHB patients. However, there were a few limitations. First, the sample size of the CHB patients was small, which may result in misperceived statistical significance. Second, the study’s cross-sectional nature failed to detect fluctuations in different clinical phases. Third, no parallel cccDNA measurement was done in this study, which has previously been shown to correlate more strongly with qHBsAg [30]. Finally, we were not able to rule out the role of qHBsAg in different HBV genotypes, clinical phases, the extent of liver damage, or patients on therapy.

## Conclusions

HBV-DNA tests are expensive and require a separate molecular biology laboratory setting and technical expertise. On the contrary, qHBsAg can be estimated in a much-simplified way, in a fully automated manner, in any peripheral laboratory setting. Periodic estimation of qHBsAg can reflect the actual viral replicability or persistence in hepatocytes. We concluded that qHBsAg levels exhibit a good correlation with HBV-DNA load and can be used as an adjunct to other routine virological investigations. Besides, in peripheral rural areas, where molecular biology laboratory facilities are unavailable, qHBsAg can be a potential alternative for predicting HBV-induced disease progression earlier.
